# One-step synthesis of OH-TiO_2_/TiOF_2_ nanohybrids and their enhanced solar light photocatalytic performance

**DOI:** 10.1098/rsos.172005

**Published:** 2018-06-06

**Authors:** Chentao Hou, Wenli Liu

**Affiliations:** College of Geology and Environment, Xi'an University of Science and Technology, Xi'an 710054, People's Republic of China

**Keywords:** TiOF_2_, OH-TiO_2_/TiOF_2_, MB, solar light, TiO_2_, network

## Abstract

TiO_2_/TiOF_2_ nanohybrids were quickly synthesized through a hydrothermal process using titanium n-butoxide (TBOT), ethanol (C_2_H_5_OH) and hydrofluoric acid as precursors. The prepared nanohybrids underwent additional NaOH treatment (OH-TiO_2_/TiOF_2_) to enhance their photocatalytic performance. In this paper, the mechanism of NaOH affecting the pathway of transformation from TBOT (Ti precursor) to TiO_2_ nanosheets was discussed. The synthesized TiO_2_/TiOF_2_ and OH-TiO_2_/TiOF_2_ were characterized by field emission scanning electron microscopy (FE-SEM), high-resolution transmission electron microscopy (HRTEM), X-ray diffraction pattern (XRD), Fourier infrared spectroscopic analysis (FT-IR), Photoluminescence (PL) emission spectra and UV–visible diffuse reflection spectra (UV–vis DRS). The photocatalytic activity and stability of synthesized samples were evaluated by degradation of methylene blue (MB) under the simulated solar light. The results showed that a larger ratio of TiO_2_ to TiOF_2_ in TiO_2_/TiOF_2_ and OH-TiO_2_/TiOF_2_ nanohybrids could allow for even higher MB conversion compared with only TiO_2_ nanosheets. NaOH treatment can wash off the F ions from TiOF_2_ and induce this larger ratio. The highest efficiency of MB removal was just above 90% in 1 h. Lower electron–hole pairs recombination rate is the dominant factor that induces the photocatalytic performance enhancement of TiO_2_/TiOF_2_ nanohybrids. The synthesized OH-TiO_2_/TiOF_2_ nanohybrids exhibit great potential in the abatement of organic pollutants.

## 1. Introduction

As one of the most important materials, TiO_2_ has been widely used as a promising catalyst due to its lack of toxicity, high stability and easy preparation. However, it has intrinsic faults of a wide energy band gap (3.1–3.2 eV, meaning it only responds to UV light) and high electron–hole recombination, which hinders its use under solar or visible light [1–4]. Many studies examining TiO_2_ has been devoted to reducing its energy band gap or photoelectron–hole separation [5–11].

Recently, Wen *et al.* [12] proposed a new visible light-driven TiOF_2_ photocatalyst for H_2_ evolution. Furthermore, Wang *et al.* [13] discovered a type of TiOF_2_ photocatalyst that possesses proper activity and strong durability in photocatalytic degradation of rhodamine B and 4-chlorophenol under visible light, although its photocatalytic performance is not ideal. Heterostructured photocatalysts have attracted increasing attention during the past few years. The electronic assembling of different nanomaterials possessing dissimilar crystal structure and band edge positions allows the complete utilization of incident photons, while in-built electric fields at the interface assist effective charge carrier separation and induce excellent performance in terms of photocatalytic activity [5–11]. However, only a few investigations on the TiO_2_/TiOF_2_ nanocomposites with heterostructures have been performed so far. Zhao *et al.* [14] reported on a Pd@TiO_2_/TiOF_2_ photocatalyst made of TiO_2_ shell and TiOF_2_ core (labelled as TiO_2_/TiOF_2_) and further improved its performance by loading Pd nanoparticles onto the surfaces of TiO_2_/TiOF_2_ heterostructure, although its synthesis process is complex.

Among the typical synthesis methods, titanium (IV) butoxide and hydrofluoric acid (HF) are the most common precursors to provide anatase TiO_2_ nanosheets with exposed facets [15–22]. In these papers, TiOF_2_ was sometimes characterized by X-ray diffraction with a peak at 2*θ* = 23.9°, which occurs independently of the main (101) peak of anatase TiO_2_ [17–22]. Almost all of these studies noted that TiOF_2_ is the intermediate compound during the transformation of Ti^4+^ to titanium nanosheets (TiO_2_) and aimed to suppress its appearance to obtain pure TiO_2_ nanosheets [17–22]. A few of these studies paid attention to the photocatalytic activity of TiOF_2_ or TiO_2_/TiOF_2_ systems, which performed more poorly when compared with (001)TiO_2_ under UV or solar light [20–22]. For example, Lv *et al*. found that when TiOF_2_ was calcined at 300°C, anatase TiO_2_ and TiOF_2_ were both observed, although the TiOF_2_ and TiO_2_/TiOF_2_ samples showed poor photocatalytic activity compared with (001) TiO_2_ under UV light for reactive brilliant red X3B [20]. Huang *et al*. also found that TiOF_2_ has poor photocatalytic performance, although the performances of TiO_2_/TiOF_2_ systems were enhanced with an increased proportion of TiO_2_ [21]. Yua *et al*. discussed the appearance of TiOF_2_ and TiO_2_ under different F/Ti atomic molar ratios, although they aimed to find the optimal ratios of exposed (101) and (001) facets of TiO_2_. However, they did not mention the role of TiOF_2_ in the TiO_2_/TiOF_2_ system [22]. Zhang *et al*. found that the mixture of (001)TiO_2_ and TiOF_2_ showed better photocatalytic activity for Rhodamine B (RhB) under simulated sunlight when they studied the reaction products of titanium butoxide and hydrogen fluoride. However, this did not show such good performance on MB for a reaction time as long as 10 h [23].

Alkali modification of catalysts has also been proven to be an effective way to enhance the photoactivity with methane dehydroaromatization, cumene cracking and CO oxidation [23–27]. For example, Han *et al*. found that alkali modification could form more hydroxyl groups on Au catalysts to enhance their catalytic activity [24]. Alkali-modified ZSM-5 zeolite also showed enhanced catalytic performance due to the formation of additional mesopores and the improvement in mass transfer and reaction kinetics [25,26]. NaOH-modified Pt/TiO_2_ also showed enhanced performance in terms of the oxidation of formaldehyde under room temperature [27]. These studies have encouraged the modification of TiO_2_/TiOF_2_ by NaOH to enhance its catalytic performance.

In this study, TiO_2_/TiOF_2_ nanohybrids were synthesized and (001) TiO_2_ was obtained through NaOH washing to remove the surface fluorine ions. NaOH was also used to modify the TiO_2_/TiOF_2_ nanohybrids to enhance their performance. This has not been investigated before. TiO_2_/TiOF_2_ nanohybrids even showed superior catalytic photoactivity towards methylene blue (MB) degradation under simulated sunlight for the first time. The possible mechanism of the TiO_2_/TiOF_2_ system in enhancing photocatalytic performance was also discussed.

## 2. Material and methods

### 2.1. Materials

Tetrabutyl titanite (TBOT) was purchased from Fu Chen Chemical Reagent Factory, Tianjin, China. HF was purchased from Xilong Chemical Industry Co Ltd, Sichuan, China. Sodium hydroxide (NaOH) and ethanol (C_2_H_5_OH) were purchased from Fuyu Fine Chemical Co., Ltd. Tianjin, China. Terephthalic acid, potassium iodide and p-benzoquinone were purchased from Shanghai Macklin Biochemical Co., Ltd., Shanghai, China. All reagents were A.R. grade and used without further purification. Ultra-pure water (18.2 MΩ•cm) was used as the water in all experiments.

### 2.2. Synthesis of TiO_2_/TiOF_2_ nanohybrids

A total of 15.2 ml of ethanol was added into 17.6 ml of TBOT, which was named solution A. Another 15.2 ml of ethanol and 5 ml of HF were added into 90 ml of ultra-pure water, which was named solution B. Solution A was dropped into solution B under medium-speed magnetic stirring at room temperature for 1.5** **h to obtain a faint yellow sol. After this, the sol was transferred into a 200-ml Teflon-lined stainless steel autoclave. The autoclave was placed into an oven, which was maintained at 100°C for 0.5, 1, 1.5 and 2** **h before being cooled to room temperature naturally to obtain a white precipitate. Ultra-pure water and C_2_H_5_OH were used to wash the precipitates several times to reach a pH of 7, before the precipitates were dried at a temperature of 100°C. The prepared samples were denoted as S0.5, S1, S1.5 and S2. The samples were then dispersed in 80 ml of 5 M NaOH solution for 30 min, before being washed with ultra-pure water and C_2_H_5_OH to reach a pH of 7. Finally, these samples were dried at 100°C for 12** **h. The prepared samples were denoted as OH-S0.5, OH-S1, OH-S1.5 and OH-S2.

### 2.3. Characterization

The crystal structure was analysed by a XD-2 X-ray diffractometer (Beijing Purkinje, China) with Cu-Kα radiation. The morphology was examined by FE-SEM (JEOL JSM6700, Japan) equipped with EDS to probe elemental analysis and high-resolution transmission electron microscopy (HRTEM; Tecnai G2 F20, FEI, USA) using an accelerating voltage of 200** **kV. Specific surface area and porosimetry were measured using Micromeritic TriStar II 3020 micrometrics (Micromeritics, USA), and the Brunauer–Emmett–Teller (BET) method was used to calculate the surface area (S_BET_). Fourier transform infrared (FT-IR) spectra were recorded using a TENSOR27 (Bruker, Germany). The optical properties were determined by UV–vis diffuse reflectance spectroscopy (UV–vis DRS; Shimadzu 2600, Japan). Photoluminescence (PL) emission spectra were measured at room temperature with a fluorescence spectrophotometer (Hitachi F-2700, Japan) using a 325-nm line with an Xe lamp. X-ray photoelectron spectroscopy (XPS) took place under an ultra-high vacuum (10** **Pa) at a pass energy of 100** **eV on a Escalab 250 Xi system (ThermoFisher, USA) equipped with a dual X-ray source by using a Al K Alpha anode and a hemispherical energy analyser. All binding energies were calibrated with contaminant carbon (C1** **s = 284.6** **eV) as a reference.

### 2.4. Photocatalytic experiments

Photocatalytic activity was measured by degradation of MB. A total of 0.015** **g of the catalyst was dispersed in a 150-ml double-layered quartz reactor containing 100 ml of a 10.0** **mg l^−1^ MB solution. Cooling water was introduced into the interlayer of the quartz reactor to maintain the solution at room temperature. A Jiguang-300 W Xe lamp (simulating solar light) was located 30** **cm away from the MB solution. A JB-420 cutoff filter was chosen to filter off light less than 420** **nm to simulate visible light. The solution was magnetically stirred for 0.5** **h in the dark to obtain the adsorption–desorption equilibrium, before the Xe lamp was turned on to start the degradation. At time intervals of 0.5** **h, about 4.0 ml of the solution was extracted and centrifuged at a speed of 10 000** **r.p.m. to remove catalysts. After this, the MB concentration was analysed with a Purkinje UV1901 UV–vis spectrophotometer at 665** **nm. The photocatalyst was separated from the MB solution, before another run was started to investigate the durability of catalysts.

### 2.5. Radical-scavenging experiments

Radical-scavenging experiments were performed to ascertain the main active species in the photocatalytic process. Terephthalic acid (3** **mmol l^−1^), potassium iodide (3** **mmol l^−1^) and p-benzoquinone (3** **mmol l^−1^) were added to a mixed solution containing 15** **mg of OH-TiO_2_ and 100 ml of 10** **mg l^−1^ of MB solution, respectively, while MB was degraded as a control.

## 3. Results

### 3.1. The formation mechanism of TiOF_2_, TiO_2_/TiOF_2_ and TiO_2_

**Figure 1.**
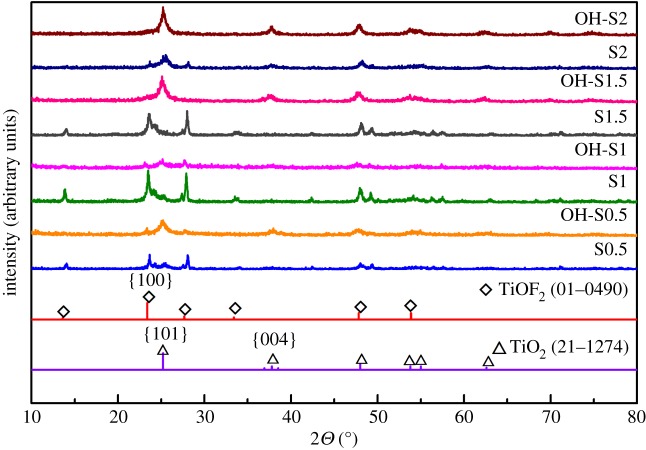
XRD patterns of prepared samples of S0.5, S1, S1.5, S2, OH-S0.5, OH-S1, OH-S1.5 and OH-S2.

The XRD patterns of the prepared samples were obtained under different experimental conditions (figure 1). All S0.5, S1, S1.5 and S2 samples showed diffraction peaks at 23.42° for TiOF_2_ (100) facet and at 25.3° for TiO_2_ (101) facet, indicating that the TiOF_2_ (JCPDS No. 01–0049) and anatase TiO_2_ (JCPDS No. 21–1272) phases coexist. Therefore, the S0.5, S1 and S1.5 samples are TiO_2_/TiOF_2_ nanohybrids. It also can be seen that with a longer reaction time, the ratio of peak height of (100) for TiOF_2_ and TiO_2_ for (101) changed from 0.44 to 4.4–4.5 and then to 0.55. This indicates that TiO_2_ existed at the beginning, before the level decreased and was dominated by other compounds, then appeared and dominated again. This result is consistent with previous research [22]. After being washed with NaOH, the peak at 23.42° for (100) facet of TiOF_2_ decreased and the peak at 25.3° for TiO_2_ (101) facet increased, indicating an increase in the TiO_2_/TiOF_2_ ratio with a longer reaction time. For OH-S1.5 and OH-S2, the peak at 25.3° for TiO_2_ (101) facet increased, indicating an increase in TiO_2_/TiOF_2_ ratio with a longer reaction time. For OH-S1.5 and OH-S2, the TiOF_2_ complex disappeared. The (004) facet of TiO_2_ even emerged, which was named the (001)-faceted TiO_2_ existence. It seems that OH in NaOH can exchange with F ions in the crystal lattice of TiOF_2_, converting it into (001)-faceted TiO_2_ nanosheets [14,18,22].

In summary, the chemical reactions of the formation of titanium nanosheets can be proposed. The first step is the hydrolysis reaction. The reaction of TBOT to form Ti(OH)_4_ is shown in equation (3.1). Secondly, in equation (3.2), Ti(OH)_4_ can react with HF to produce TiOF_2_ through water condensation [28,29]. Finally, TiOF_2_ can react with NaOH to form TiO_2_, as shown in equation (3.3) [30].

3.1 $$\begin{array}{rl} \mathrm{Ti}{\left({\mathrm{OC}}_{4}H_{9}\right)}_{4}+H_{2}O & \to \mathrm{Ti}{\left(\mathrm{OH}\right)}_{4}+4(C_{4}H_{9}\mathrm{OH})\;(\mathrm{hydrolysis}), \end{array}$$

3.2 $$\begin{array}{rl} \mathrm{Ti}{\left(\mathrm{OH}\right)}_{4}+2\mathrm{HF} & \to {\mathrm{TiOF}}_{2}+3H_{2}O\;(\text{water condensation}) \end{array}$$

3.3 $$\begin{array}{rl} \;\text{and}\;\;{\mathrm{TiOF}}_{2}+2\mathrm{NaOH} & \to {\mathrm{TiO}}_{2}(\mathrm{anatase})+2\mathrm{NaF}+H_{2}O\;(\text{in situ}\;\mathrm{transformation}). \end{array}$$

This study provides a simple conversion method of TiOF_2_ to TiO_2_ in addition to the calcination of TiOF_2_ [20,21].

### 3.2. Morphology analysis

**Figure 2.**
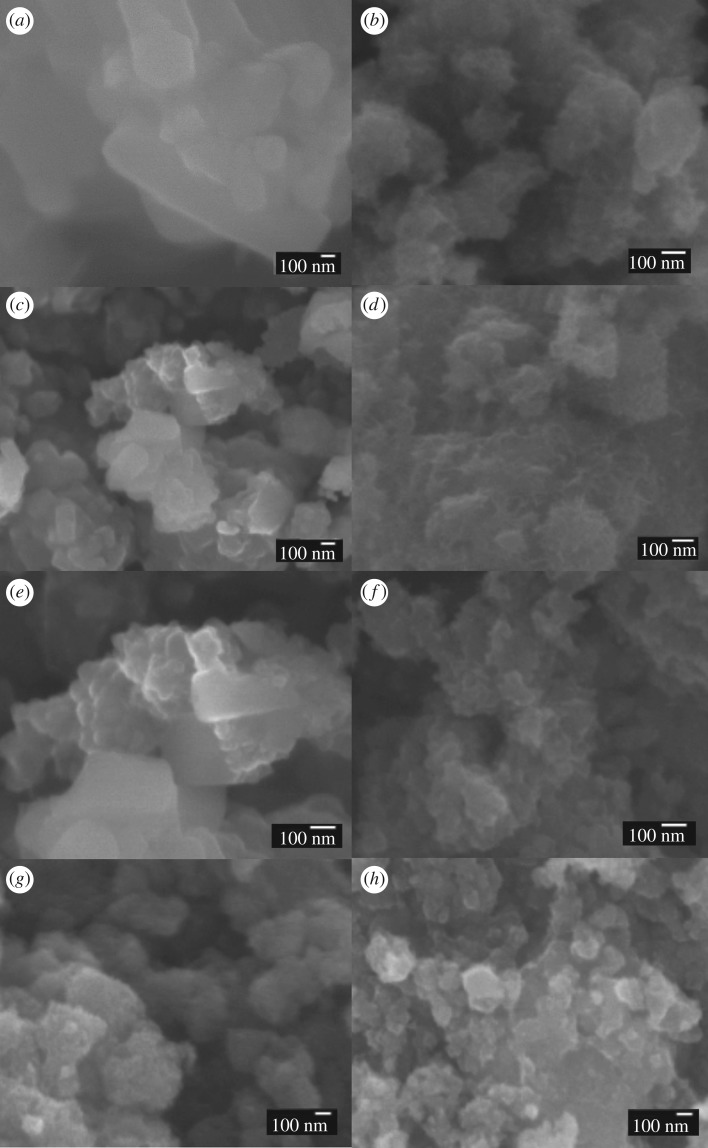
SEM of (*a*) S0.5, (*b*) OH-S0.5, (*c*) S1, (*d*) OH-S1, (*e*) S1.5, (*f*) OH-S1.5, (*g*) S2 and (*h*) OH-S2.

**Figure 3.**
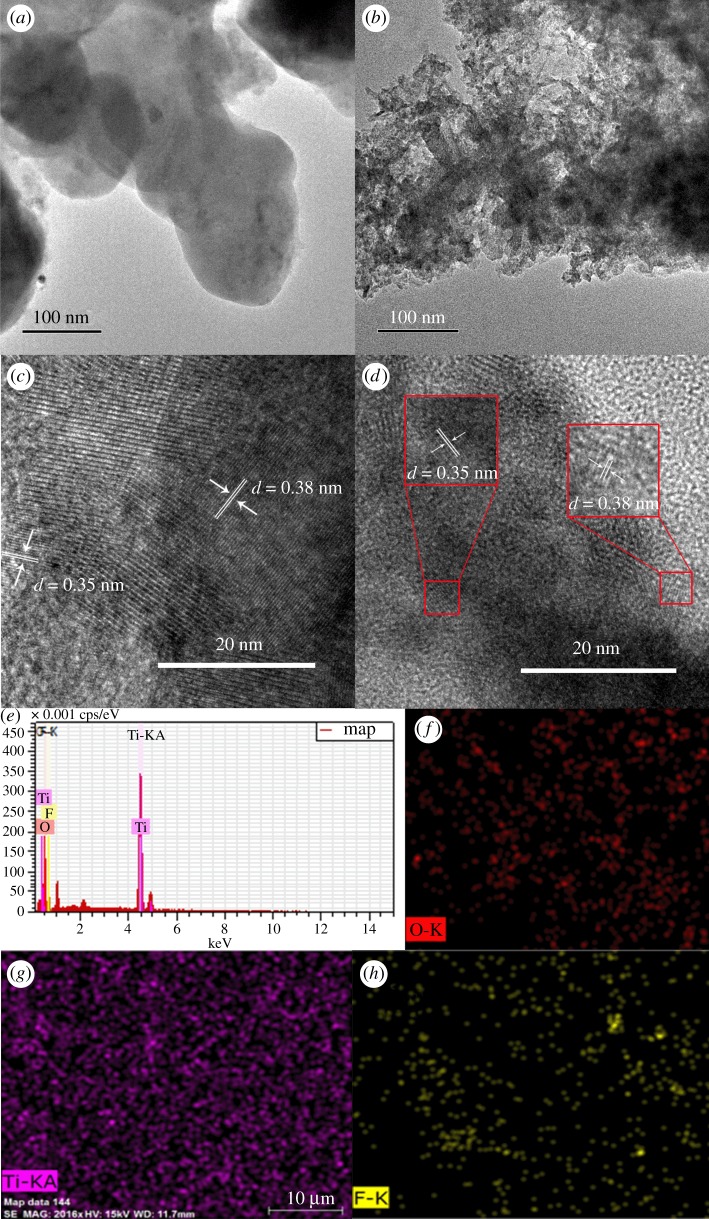
HRTEM and EDS spectra of S0.5 and OH-S0.5 samples: (*a*) TEM of S0.5, (*b*) TEM of OH-S0.5, (*c*) HRTEM of S0.5, (*d*) HRTEM of OH-S0.5, (*e*) EDS of OH-S0.5, and (*f*–*h*) EDS elemental mapping of OH-S0.5.

The morphology of the prepared samples was characterized by FE-SEM and high-resolution transmission electron microscope (HRTEM) in figures 2 and 3. The SEM and HRTEM images shown in figure 2 and figure 3*a,b*, respectively, were used to obtain the morphology of photocatalysts. The S0.5, S1 and S1.5 samples exhibit mixtures of two types of crystals connected together, which correspond to TiOF_2_ and TiO_2_ according to the XRD results in figure 1. By contrast, S2 shows a nano-network look, corresponding to TiO_2_. The OH-S0.5, OH-S1 and OH-S1.5 samples exhibited a nano-network look after reaction with NaOH, permitting light scattering inside the catalyst and enhancing its absorption in light.

As also can be seen in figure 3*c,d*, the lattice fringes of 0.19 nm, 0.235 nm and 0.352 nm were assigned to the (200) (001) and (101) planes of TiO_2_, respectively [18,22,30]. The lattice fringes of 0.38 nm were assigned to the (100) planes of TiOF_2_ [12,13]. This also indicates that the S0.5 and OH-S0.5 samples are nanohybrids of TiO_2_ and TiOF_2_, which is consistent with the XRD and SEM results. The EDS spectrum (figure 3*e*) shows that OH-S0.5 consists of Ti, O and F elements. The uniform distribution of Ti, O and F can be seen from the EDS elemental mapping (figure 3*f–h*), which suggests the successful formation of a heterojunction.

### 3.3. S_BET_ and pore-size distribution

**Figure 4.**
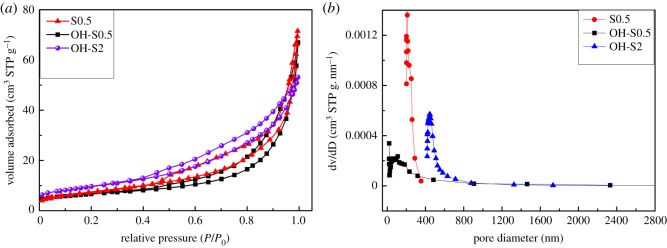
(*a*) Nitrogen adsorption–desorption isotherms and (*b*) the corresponding pore-size distribution curves of S0.5, OH-S0.5 and OH-S2.

Figure 4 shows the nitrogen adsorption–desorption isotherms and the corresponding pore-size distribution curves of prepared samples. Considering the BET data in electronic supplementary material, table S1, the S_BET_ and pore volume of S2 can reach as high as 59.3 m^2^·g^−1^ with a pore size of 62.5 nm, while the S_BET_ of S0.5 and OH-S0.5 are significantly lower with values of 23.6 and 27.21 m^2^·g^−1^, respectively, and pore sizes of 122.6 and 14.49 nm, respectively. After NaOH washing, the S_BET_ of OH-S0.5 was a bit lower than that of S0.5, but the pore size was dramatically decreased.

### 3.4. FT-IR analysis

**Figure 5.**
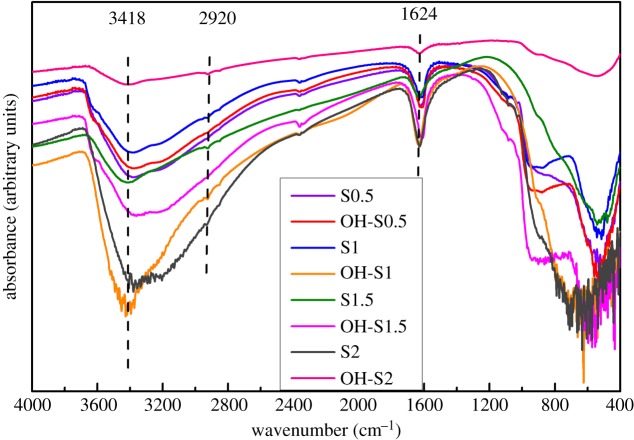
FT-IR spectra of S0.5, S1, S1.5, S2, OH-S0.5, OH-S1, OH-S1.5 and OH-S2.

As shown in figure 5, broad absorptions centered around 3418 and 2920 cm^−1^ as well as the weak sharp absorption band centred around 1624 cm^−1^ were attributed to the hydroxyl free radicals, associated hydrogen bonds and absorption water. The OH and water in all NaOH-reacted samples are all stronger than those modified before, with the exception of OH-S2. For the OH-S2 sample, the OH from the broad absorptions centred around 3418, 2920 and 1624 cm^−1^ sharply decreased. According to XRD results, the S2 and NaOH-reacted samples are all TiO_2_/TiOF_2_ nanohybrids, except OH-S2. It was concluded that TiO_2_/TiOF_2_ nanohybrids can adsorb more OH to enhance photocatalytic performance [31–37].

### 3.5. XPS analysis

**Figure 6:**
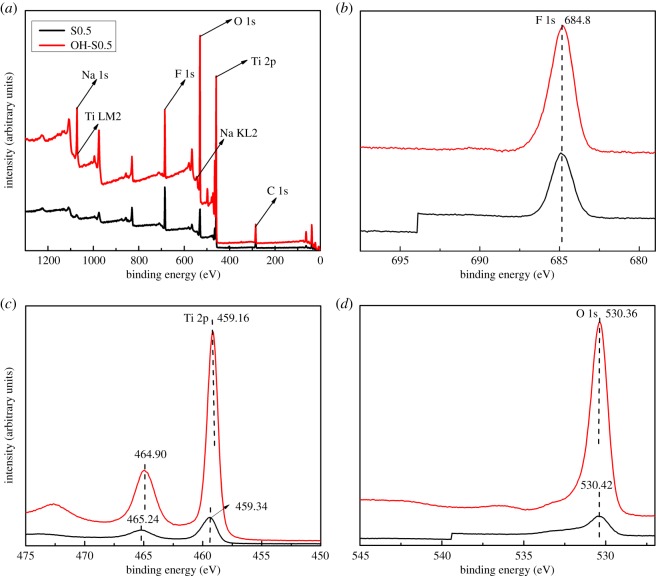
High-resolution XPS survey spectra for (*a*) all samples, (*b*) F 1s, (*c*) Ti 2p and (*d*) O 1s of the S0.5 and OH-S0.5 samples.

In figure 6, XPS survey spectra of the prepared samples of S0.5 and OH-S0.5 are presented. As observed in figure 6*a*, all the samples have sharp photoelectron peaks at binding energies of Ti 2p, O 1s and F 1s. Another sharp photoelectron peak appears in all samples at the binding energy (BE) of 285 eV (C 1s) due to the contamination of the XPS instrument itself [23,38–41]. The F 1s BE of 684.8 eV in this spectrum (figure 6*b*) corresponds to that of F adsorbed on TiO_2_, while there was no sign of F ions in the lattice (BE = 688.5 eV) [38,39]. The measured binding energies of Ti 2p3/2 and Ti 2p1/2 (figure 6*c*) for S0.5 were 459.3 and 465.2 eV, respectively. The main sharp peaks of Ti 2p3/2 were assigned to Ti^4+^ in TiO_2_ [23,39–41]. As compared to S0.5, the binding energies of OH-S0.5 shifted to 459.16 and 464.9 eV, respectively. One possible explanation is that TiO_2_ is partially reduced into Ti^3+^ [23,41]. As Ti^3+^is more hydrophilic than Ti^4+^, oxygen and water molecules are easily adsorbed on the surface of TiO_2_, which consequently facilitates the formation of surface OH groups. The measured BE of O 1s (figure 6*d*) was 530.42 eV, while this shifted to 530.36 eV in OH-S0.5. This can be explained by OH-S0.5 possibly having many oxygen vacancies that are generated at the interface of TiO_2_/TiOF_2_. Oxygen from the gas phase could dissociate and become adsorbed on such defects, thus resulting in a decrease in binding energies of O 1s to TiO_2_ lattice oxygen (Ti−O−Ti) [23]. So it can be concluded that NaOH treatment can induce more surface OH groups and oxygen vacancies at the interface of TiO_2_/TiOF_2_, which would enhance the photocatalytic performance of TiO_2_/TiOF_2_ nanohybrids.

### 3.6. UV–vis DRS analysis

**Figure 7.**
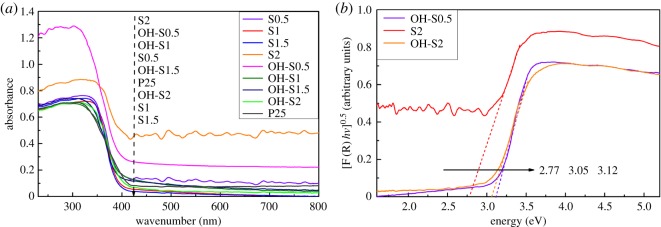
(*a*) UV–vis absorption spectroscopy and (*b*) band gap of prepared TiO_2_ /TiOF_2_ nanohybrids and TiO_2_ samples.

Figure 7 shows the UV–vis absorption spectroscopy and band gap of the prepared TiO_2_/TiOF_2_ nanohybrids and TiO_2_ samples. It can be seen that S2 and OH-S0.5 have a greater increase in adsorption in both the range of UV and visible light compared with other samples. By contrast, they contain more TiO_2_ compared to S0.5, which corresponds to the XRD results. While the OH-S2 sample has weaker adsorption than S2, it becomes TiO_2_ after NaOH washing. Therefore, a larger ratio of TiO_2_/TiOF_2_ causes the stronger light adsorption. The band gaps of S2, OH-S2 and OH-S0.5 are 2.77, 3.05 and 3.12 eV, respectively, which are lower than that of P25 (3.2 eV). This indicates that they are more easily excited by visible light.

### 3.7. PL analysis

**Figure 8.**
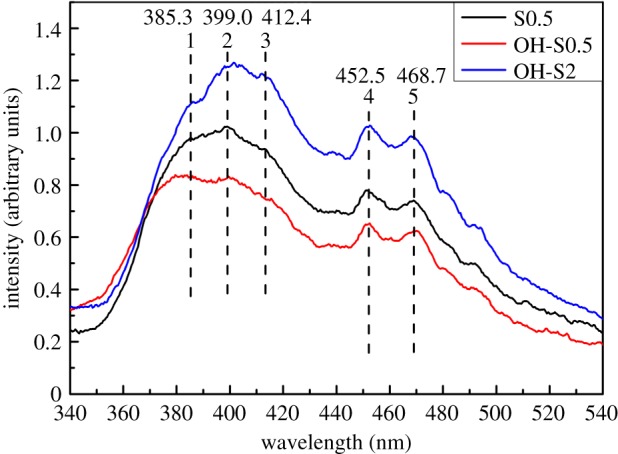
PL spectra of S0.5, OH-S0.5 and OH-S2 samples.

PL emission spectra were used to investigate the efficiency of charge carrier trapping, immigration and transfer as well as to understand the fate of electron hole pairs in catalysts (figure 8). Six peaks were observed in the spectra. The broad emission bands centred at 385.3 nm (peak 1), 399.0 nm (peak 2) and 412.4 nm (peak 3) were ascribed to form the boundaries of exciton emission due to the trapping of free excitons by titanite groups near defects [42]. The long wavelength range of 452.5–468.7 nm (peaks 4 and 5) is attributed to the oxygen vacancy with two trapped electrons. Oxygen vacancy sites are important for the formation of superoxide (O_2_•−) and hydroxyl (•OH) radicals for photocatalytic degradation. A lower PL intensity also indicates a lower recombination rate of electron–hole pairs and higher separation efficiency, thus representing higher photocatalytic activity [43].

### 3.8. Catalytic activities of TiO_2_/TiOF_2_ and TiO_2_ photocatalysts

**Figure 9.**
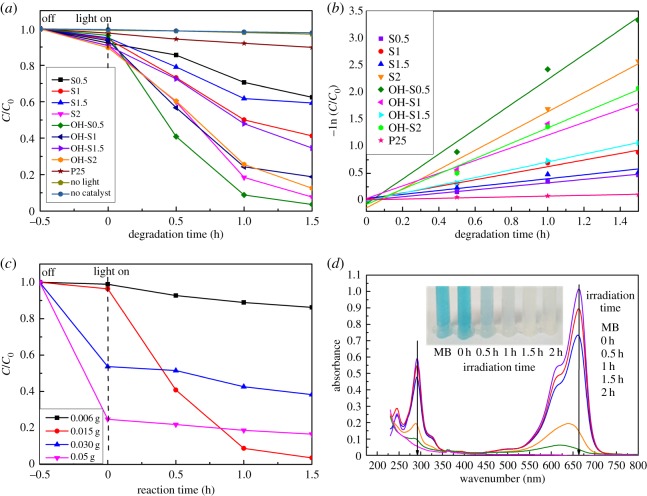
Solar light photocatalytic properties of the prepared samples, P25 and UV–vis spectra of MB with irradiation time: (*a*) comparison of solar light-sensitized degradation of MB in the suspension of samples; (*b*) the reaction rate of all samples; (*c*) the effect of different amounts of catalysts on the solar light photocatalytic properties of the prepared OH-S0.5 samples; and (*d*) UV–vis absorption spectral changes of MB with solar light irradiation time by OH-S0.5.

Figure 9*a* shows the solar light photocatalytic properties of the prepared samples and P25. Figures 9*a* and S1 showed that with 0.015 g/100 ml of catalysts, the decrease in MB was very small in the first 0.5 h in dark and in light without a catalyst, indicating that this decrease in MB was a photocatalytic process with all samples being activated in solar or visible light. It also shows that the S2 and OH-S0.5 samples can cause almost complete decomposition of MB in about 1.5 h with better photocatalytic performance than all of the other samples. By contrast, P25 performed poorly compared to TiO_2_/TiOF_2_ and (001) TiO_2_. All NaOH-treated nanohybrids showed higher performance than untreated ones except the pure (001)-facet TiO_2_ from S2.

The activity arrangement order was consistent with the TiO_2_/TiOF_2_ ratio. A larger ratio of TiO_2_/TiOF_2_ resulted in better photocatalytic activity. This result is consistent with Yua *et al.*'s research [22]. It can be explained by the better charge separation capability of the TiOF_2_–TiO_2_ mixed phase, which reduces the recombination rate of electron–hole pairs. However, a high amount of TiOF_2_ phase in the TiO_2_ nanosheets would decrease the photoactivity due to poor photoactivity of TiOF_2_.

The reaction rate of all of the samples is shown in figure 9*b*. The data were fitted with the first-order reaction equation as follows:

3.4 $$\ln\;\left(\frac{C_{0}}{C}\right)=kt,$$

where *t* is the reaction time, $C_{0}$ is the concentration of RhB at time 0, *C* is the concentration of RhB at time *t*, and *k* is the reaction rate constant. Figure 9*b* shows that P25 had a rate constant of only 0.07 h^−1^, indicating poor photocatalytic performance. The calculated rate constants are 1.4 and 2.3 h^−1^ for S2 and OH-S0.5, respectively. The OH-S0.5 sample shows the best performance among all the photocatalysts, with a degradation rate that is much higher than that of the P25, TiO_2_/TiOF_2_ and TiO_2_ samples. This excellent performance could be mainly attributed to its stronger light adsorption and TiO_2_/TiOF_2_ combination.

The catalytic abilities of 0.006 g/100 ml, 0.030 g/100 ml and 0.050 g/100 ml (OH-S0.5) are also shown in figure 9*c*. For the catalytic amount of 0.015 g/100 ml, the photocatalytic degradation rate of MB was the strongest and had the best degradation effect (figure 9*c*). When the content of OH-S0.5 was increased, its adsorptive capacity increased but photocatalytic degradation performance decreased (figure 9*c*).

Figure 9*d* shows the UV–vis absorption spectral changes of the MB solution. According to previous research, the discoloration of MB can be caused in two ways: the oxidative degradation and the two-electron reduction to leuco-MB, which can be detected by the UV–vis absorption at 256 nm [44,45]. Figure 9*d* shows that there is a blue-shift from 665 to 625 nm with an absorbent peak emerging at 256 nm after the spectral change of MB with the irradiation time by OH-S0.5. This means that a reductive conversion to leuco-MB exists in the degradation path of MB.

### 3.9. Radical-scavenging experiments

**Figure 10.**
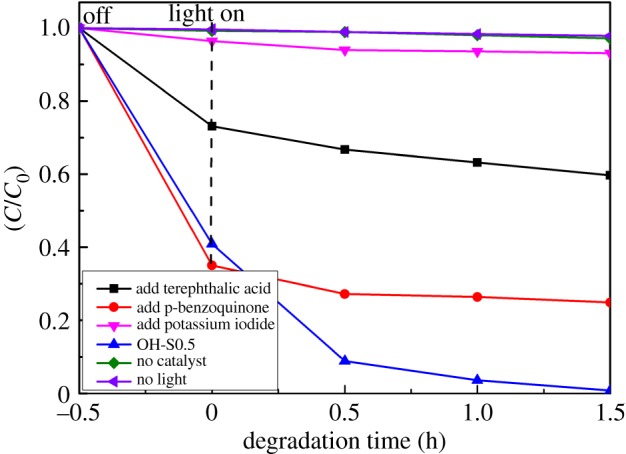
Photocatalytic degradation of MB in the presence of different scavengers over OH-S0.5 under simulated solar light irradiation.

Radical-scavenging experiments were performed to complete an in-depth study of the photocatalytic degradation mechanism. The reactive species were detected through trapping experiments of hydroxyl radicals (•OH), holes (h^+^) and superoxide radical anions (O_2_•−) by introducing terephthalic acid [46], potassium iodide [46] and p-benzoquinone [47]. Terephthalic acid can combine with the hydroxyl radicals (•OH), potassium iodide can combine with holes (h^+^), and p-benzoquinone can combine with the superoxide radical (O_2_•−), to decrease the activity of the catalyst [46,47]. The effects of a series of scavengers on the photocatalytic oxidation towards the MB dye over the photocatalysts are shown in figure 10. Under simulated sunlight illumination, the percentage of MB loss decreased most rapidly after the addition of potassium iodide, indicating h^+^ was the main active species in the photocatalytic process. When terephthalic acid and p-benzoquinone were added, this also reduced activity, which implied that •OH and O_2_•− radicals also played a role in the photooxidation of MB. The order of importance of the active species is h^+^, •OH and O_2_•−.

## 4. Discussion

TiOF_2_ photoactivity was quite low, nearly 150 times lower than P25 in the X3B dye degradation experiment [36]. Lv *et al*. reported that surface fluorination can greatly enhance the photocatalytic activity of TiO_2_ due to the formation of free •OH radicals, which are highly reactive [37]. In this research, a combination of TiO_2_ and TiOF_2_ demonstrated even higher photoactivity performance in MB degradation compared with the pure TiO_2_ nanosheets. In order to further understand the reason for the enhanced photocatalytic performance, a possible mechanism of charge separation and transfer on the surface of TiO_2_/TiOF_2_ nanohybrids is proposed. The conduction band (*E*_c_) and valence band (*E*_v_) potentials of TiO_2_ and TiOF_2_ at the point of zero charge can be calculated by the following empirical equation [11,14,44]: *E*_c_ = *χ* − *E*_0_ − 0.5 *E*_g_, where *E*_c_ is the energy of the conduction band, *X* is the bulk Mulliken electronegativity of the compound, *E*_0_ is the energy of free electrons on the hydrogen scale (about 4.5** **eV), and *E*_g_ is the band gap energy of the semiconductor. The *x*-values for TiO_2_ and TiOF_2_ are approximately 5.8 and 7.3** **eV, respectively. The energy gap (*E*_g_) was estimated from the intercept of the tangent in the plots of ${\left(\alpha h\nu \right)}^{1/2}$ versus photon energy (hν), which is shown in figure 7*b*. The *E*_g_ of TiO_2_ and TiOF_2_ were evaluated to be 3.2** **eV and 2.94** **eV, respectively.

The position of the valence band edge (*E*_v_) is determined by the following equation:

4.1 $$E_{v}=E_{c}+E_{g}.$$

**Figure 11.**
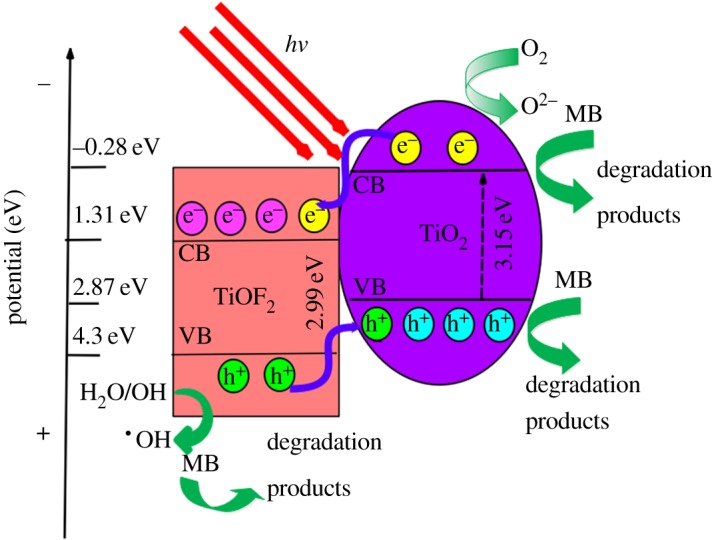
Schematic diagram for the conduction and valence bands of TiO_2_ and TiOF_2_, and the degradation route of MB under solar light irradiation.

The calculated *E*_c_ and *E*_v_ of TiO_2_ and TiOF_2_ are shown in the electronic supplementary material, table S2. The *E*_c_ edge potential of TiO_2_ (–0.3** **eV) is more active than that of TiOF_2_ (1.5** **eV). Hence, the photogenerated electrons on the TiO_2_ surface have a strong capability for moving onto the surfaces of TiOF_2_ via the interface transfer pathway. Similarly, photogenerated holes on the TiOF_2_ surface migrate to TiO_2_ under the driving force of the *E*_v_ edge potentials (figure 11). As a result, the electron–hole recombination is reduced, which is consistent with PL analysis. Thus, under simulated solar light irradiation, more h^+^ radicals can react with H_2_O or OH^−^ to produce **•**OH, while more electrons can react with the dissolved oxygen molecules to yield superoxide radical anions (O_2_**•**−) [48,49]. MB can also be decomposed by the holes directly [50]. Because the photocatalytic activity of TiO_2_ is better than that of TiOF_2_, a larger ratio of TiO_2_ /TiOF_2_ (S2 and OH-S0.5) results in better photocatalytic activity. NaOH treatment can induce more F ions being disconnected from the surface of TiOF_2_, which means a larger ratio of TiO_2_/TiOF_2_ (XRD results). Furthermore, the presence of OH bonds on the surface of the photocatalyst (FT-IR analysis) and more oxygen vacancies (XPS and PL analysis) also induce the production of more **•**OH with enhancement of the photocatalytic performance. This can explain why NaOH-treated nanohybrids showed greater performance than untreated ones. OH-S0.5 showed the best photocatalytic activity, even better than S2; this may be attributed to its lower recombination rate of electron–hole pairs introducing more h^+^, and more Ti^4+^ being reduced into Ti^3+^, because its OH, S_BET_ and absorption of light are not the largest according to FT-IR and UV–vis DRS analysis. So a lower electron–hole pairs recombination rate is the dominant factor that induces the photocatalytic performance enhancement of TiO_2_/TiOF_2_ nanohybrids. This was in accord with the radical-scavenging experiments**.**

## 5. Conclusion

In summary, an easy one-step hydrothermal route to synthesize TiO_2_/TiOF_2_ and OH-TiO_2_/TiOF_2_ hybrids has been demonstrated. The introduction of NaOH facilitated the conversion from TiOF_2_/TiO_2_ and induced a network structure. The prepared TiO_2_/TiOF_2_, especially the OH-TiO_2_/TiOF_2_ nanocomposite, exhibited excellent activity towards the degradation of MB under simulated sunlight irradiation. A larger ratio of TiO_2_/TiOF_2_ in TiO_2_/TiOF_2_ and OH-TiO_2_/TiOF_2_ nanohybrids could enable better performance. NaOH treatment can wash off the F ions from TiOF_2_ and induce this larger ratio. The highest efficiency of MB removal was just above 90% in 1 h. A lower electron–hole pairs recombination rate is the dominant factor that induces the photocatalytic performance enhancement of TiO_2_/TiOF_2_ nanohybrids.

Therefore, this work opens an avenue to efficiently synthesize TiO_2_/TiOF_2_ through a one-step nanocomposite for the removal of organic pollutants.

## Supplementary Material

Supplementary file-One-Step synthesis of OH-TiO_2_/TiOF_2_ nanohybrids and it's enhanced solar light photocatalytic performance

## Data accessibility

The datasets supporting this article have been uploaded as part of the electronic supplementary material.
