# Supplementary material for: One-step synthesis of OH-TiO2/TiOF2 nanohybrids and their enhanced solar light photocatalytic performance
Source: R Soc Open Sci. 2018 Jun 6;5(6):172005. doi: 10.1098/rsos.172005 (PMC6030343; doi:10.1098/rsos.172005)
Supplement: Supplementary file-One-Step synthesis of OH-TiO2/TiOF2 nanohybrids and it's enhanced solar light photocatalytic performance [file rsos172005supp1.doc]

**Synthesis of OH-TiO_2_/TiOF_2_ nanohybrids and it’s enhanced solar light photocatalytic performance**

Hou Chentao, Liu Wenli

Table S1 S_BET_,Pore Volume and Pore Size of OH-S0.5,S2,OH-S2 and P25

| sample | SBET(m2·g-1) | Pore Size(nm) |
| --- | --- | --- |
| OH-S0.5 | 23.5778 | 122.5116 |
| S2 | 59.2675 | 62.5037 |
| OH-S0.5 | 27.21 | 14.49 |
| P25 | 51.8118 | 94.2160 |

Table S2 Absolute electronegativity (χ), energy band gap (Eg), calculated conduction band (Ec) and valance band edge (Ev) at the point of zero charge for TiO_2_ and TiOF_2_ semiconductors.

| Semiconductors | Absolute electronegativity (eV) | Calculated conduction band edge (eV) | Calculated valance band edge (eV) | Energy band gap Eg (eV) |
| --- | --- | --- | --- | --- |
| TiOF_2_ | 7.3 | 1.31 | 4.3 | 2.6 |
| TiO_2_ | 5.8 | -0.275 | 2.875 | 3.15 |





**Figure S1 comparison of visible-light-sensitized degradation of MB in the samples suspension**
